# m7G regulator-mediated molecular subtypes and tumor microenvironment in kidney renal clear cell carcinoma

**DOI:** 10.3389/fphar.2022.900006

**Published:** 2022-09-06

**Authors:** Mei Chen, Zhenyu Nie, Yuanhui Gao, Hui Cao, Linlin Zheng, Na Guo, Yanling Peng, Shufang Zhang

**Affiliations:** Central Laboratory, Affiliated Haikou Hospital of Xiangya Medical College, Central South University, Haikou, China

**Keywords:** m7G, kidney renal clear cell carcinoma, molecular subtype, tumor microenvironment, prognosis, drug sensitivity

## Abstract

**Background:** RNA methylation modification plays an important role in immune regulation. m7G RNA methylation is an emerging research hotspot in the RNA methylation field. However, its role in the tumor immune microenvironment of kidney renal clear cell carcinoma (KIRC) is still unclear.

**Methods:** We analyzed the expression profiles of 29 m7G regulators in KIRC, integrated multiple datasets to identify a novel m7G regulator-mediated molecular subtype, and developed the m7G score. We evaluated the immune tumor microenvironments in m7G clusters and analyzed the correlation of the m7G score with immune cells and drug sensitivity. We tested the predictive power of the m7G score for prognosis of patients with KIRC and verified the predictive accuracy of the m7G score by using the GSE40912 and E-MTAB-1980 datasets. The genes used to develop the m7G score were verified by qRT-PCR. Finally, we experimentally analyzed the effects of WDR4 knockdown on KIRC proliferation, migration, invasion, and drug sensitivity.

**Results:** We identified three m7G clusters. The expression of m7G regulators was higher in cluster C than in other clusters. m7G cluster C was related to immune activation, low tumor purity, good prognosis, and low m7G score. Cluster B was related to drug metabolism, high tumor purity, poor survival, and high m7G score. Cluster A was related to purine metabolism. The m7G score can well-predict the prognosis of patients with KIRC, and its prediction accuracy based on the m7G score nomogram was very high. Patients with high m7G scores were more sensitive to rapamycin, gefitinib, sunitinib, and vinblastine than other patients. Knocking down WDR4 can inhibit the proliferation, migration, and invasion of 786-0 and Caki-1 cells and increase sensitivity to sorafenib and sunitinib.

**Conclusion:** We proposed a novel molecular subtype related to m7G modification and revealed the immune cell infiltration characteristics of different subtypes. The developed m7G score can well-predict the prognosis of patients with KIRC, and our research provides a basis for personalized treatment of patients with KIRC.

## Introduction

m7G RNA methylation is catalyzed by the Trm8–Trm82 complex in yeast and by the METTL1–WDR4 complex in humans under the action of methyltransferases (A Alexandrov et al., 2002). m7G RNA methylation can regulate mRNA transcription, miRNA biological function, tRNA stability, nuclear processing, and 18S rRNA maturation. m7G regulators are prognostic markers of a variety of cancers. METTL1 and WDR4 are highly expressed in a variety of tumors, such as liver cancer (Chen D et al., 2021), intrahepatic cholangiocarcinoma (Dai S et al., 2021), and lung cancer (Ma et al., 2021), which is related to poor prognosis of patients. High expression of NSUN2 is associated with poor prognosis of gastric cancer (Hu J et al., 2021) and esophageal squamous cell carcinoma (Su et al., 2021). NUDT10 is a reliable prognostic marker of gastric cancer (Chen Z et al., 2021). GEMIN5, EIF4E3, and GEMIN5 can specifically bind to the m7G cap (Bradrick and Gromeier, 2009; Osborne et al., 2013; Xu et al., 2016). Meanwhile, NUDT16 can remove the m7G cap (Lu et al., 2011). AGO2 inhibits mRNA translation by binding to the m7G cap (Kiriakidou et al., 2007). EIF4E binds to the m7G cap to mediate mRNA translation and can increase the capping efficiency of coding and noncoding RNAs (Culjkovic-Kraljacic et al., 2020). EIF4E overexpression can promote cell proliferation and invasion of renal cell carcinoma (RCC) (Li et al., 2017). The knockdown of EIF3D can inhibit the progression of RCC by inducing G2/M arrest (Pan et al., 2016). Current studies only focused on the role of a single m7G regulator. However, multiple genes are involved in tumor occurrence, and the prognostic role of multiple m7G regulators has not been clarified.

Kidney renal clear cell carcinoma (KIRC), the most common histological subtype of RCC, is characterized by high heterogeneity and poor prognosis (Hsieh et al., 2017). Immunotherapy has led to significant progress in the treatment of patients with KIRC, and immune checkpoint inhibitors have been used as the first-line treatment of advanced KIRC (Bedke et al., 2021; Braun et al., 2021). However, some patients still experience spontaneous regression due to tumor immune escape, and the effect of immunotherapy still greatly varies across different patients. KIRC has a high degree of immune infiltration, with T-cell infiltration being the highest (şenbabaoğlu et al., 2016). CD8^+^ T-cell infiltration is associated with poor prognosis of KIRC (Dai Z et al., 2021; Li et al., 2020). The tumor microenvironment plays an important role in tumor biology and treatment. Understanding the characteristics of the tumor microenvironment under the mediation of m7G is of great importance for predicting the immunotherapy of patients with KIRC.

In this study, first, we performed consistent cluster analysis on 702 patients with KIRC, identified three m7G clusters, and studied the characteristics of immune cell infiltration, function, and survival among different subtypes. We classified the patients into three gene clusters in accordance with the differentially expressed genes (DEGs) among the three m7G clusters. We developed the m7G score to predict the prognosis of patients and analyzed its correlation with the tumor microenvironment, mutation, tumor mutation burden (TMB), and stemness indices. Finally, we verified the genes used for developing the m7G score by utilizing clinical samples.

## Materials and methods

### Data collection and processing

RNA-seq data [fragments per kilobase million (FPKM)] and KIRC clinical and mutation data were downloaded from the TCGA database (https://portal.gdc.cancer.gov/). FPKM was converted into transcripts per kilobase million (TPM). The GSE29609 (Edeline et al., 2012), GSE40912 (Fachel et al., 2013), and GSE172165 datasets were downloaded from the GEO database (https://www.ncbi.nlm.nih.gov/geo/). The E-MTAB-1980 (Sato et al., 2013) dataset was downloaded from the ArrayExpress database (https://www.ebi.ac.uk/arrayexpress/). The details of these cohorts are provided in Supplementary Data Sheet S1. A total of 702 samples were obtained through batch correction with the “sva” package. In the molecular signature database (http://www.gsea-msigdb.org/gsea), “7-methylguanosine” was used as the search term to obtain three m7G-related gene sets (GOMF_M7G_5_PPPN_DIPHOSPHATASE_ACTIVITY, GOMF_RNA_7_METHYLGUANOSINE_CAP_BINDING, and GOMF_RNA_CAP_BINDING), and 26 m7G regulators were acquired from these gene sets. Three m7G regulators were sourced from previous literature (Tomikawa, 2018). A total of 29 m7G regulators were used for analysis in this research (Supplementary Table S1). The m7GHub (http://180.208.58.19/m7g/index.html) contains m7G sites, a sequence-based high accuracy predictor, evaluation of the effects of m7G status mutations, and gene mutations regulated by m7G methylation (Song et al., 2020). Immune checkpoints with m7G methylation were screened from the m7GHub database.

### Identification of m7G subtypes

The unsupervised clustering analysis of 702 samples was conducted with the “ConsensusClusterPlus” package. The correlation between the groups was the lowest, and the correlation within the groups was the highest. The optimal K value was selected to obtain different subtypes.

### Enrichment analysis of DEGs

The DEGs among m7G subtypes were analyzed using the “limma” package. |Fold change| > 1 and adjusted *p*-value < 0.01 were set as the thresholds to identify DEGs. The DEGs were enriched and analyzed by using Gene Ontology (GO) and Kyoto Encyclopedia of Genes and Genomes (KEGG) with the “clusterProfiler” package.

### Development of the m7G score

Univariate Cox analysis was performed on the DEGs to obtain prognostic genes. The prognostic genes were analyzed through unsupervised clustering with the “ConsensusClusterPlus” package, and the patients were divided into different gene subtypes. The m7G score was developed after LASSO regression analysis. Its calculation formula is as follows:

m7G score=∑Expi×coefi

Here, Expi and coefi represent the gene expression values and correlation coefficients, respectively. The score of each patient was calculated in accordance with the formula, and the patients were divided into the training and testing groups at the ratio of 1:1. The patients were divided into the high- and low-risk groups in accordance with the median value of the training group. Kaplan–Meier (K–M) analysis was performed on the high- and low-risk groups with the “survival” and “survminer” packages. Receiver operator curves (ROC) were drawn with the “timeROC” package to evaluate the accuracy of the m7G score in predicting prognosis. A nomogram was constructed with the “rms” package in combination with clinicopathological variables.

### Pathway enrichment analysis

The “c2.cp.kegg.v7.4.symbols” gene set was selected, and the “GSVA” package was used to calculate the differential gene set among different subtypes. Molecular subtypes were visualized with the “ggplot2” package. “C2.cp.kegg.v7.5.symbols.gmt” in GSEA4.1.0 software was selected for analysis, and the other operation steps in this work were consistent with those in previous studies (Chen M et al., 2021).

### Immune cell infiltration in KIRC

The “estimate” package was used to run the ESTIMATE algorithm (Yoshihara et al., 2013), which was utilized to evaluate the presence of stromal cells and infiltration of immune cells in tumor samples and infer tumor purity. ssGSEA (Hänzelmann et al., 2013), CIBERSORT (Newman et al., 2015), and MCP counter algorithm (Becht et al., 2016) were applied to analyze the differences of immune cells between different subtypes, and Spearman’s correlation analysis was performed between the m7G score and immune cells.

### Correlation analysis between the m7G score and therapeutic drugs

The expression profiles of immune checkpoints in the high- and low-risk groups were analyzed with the “limma” package, and the IC_50_ of chemotherapeutic drugs (rapamycin, gefitinib, sunitinib, vinblastine, gemcitabine, lapatinib, and sorafenib) in KIRC was calculated with the “pRRophetic” package. The association between WDR4 and drug IC_50_ was analyzed using data from the GDSC database (https://www.cancerrxgene.org/).

The DEGs between high- and low-risk groups were divided into upregulated and downregulated genes and entered into the cAMP database (https://clue.io/) to obtain potential therapeutic drugs. The 2D and 3D structures of the drugs were obtained from the PubChem database (https://pubchem.ncbi.nlm.nih.gov/). A negative score indicates that the drug can be beneficial for treatment of patients in the high-risk group. Scores < −90 were used to identify associated small molecules.

### Clinical sample validation

Paired cancer and adjacent tissues were collected from 13 patients with KIRC in our hospital. The informed consent of the patients and the approval of the ethics committee of Haikou Hospital, affiliated to Xiangya Medical College of Central South University were obtained before specimen collection. The operation steps and calculation methods of quantitative real-time polymerase chain reaction (qRT-PCR) are shown in our previous research (Chen et al., 2020). cDNA was amplified with an Applied Biosystems QuantStudio 5 Real-Time PCR instrument. Primer sequences are provided in Supplementary Table S2.

### Cell culture and small interfering RNA transfection

786-0 and Caki-1 cell lines were purchased from the China Centre for Type Culture Collection (Wuhan, China) and cultured in an atmosphere of 5% CO_2_ and 95% air at 37°C. siWDR4 was designed and synthesized by RiboBio (Guangzhou, China) and transfected with Lipofectamine 3000 (Thermo Fisher, NY, United States). At 48 h after transfection, the cells were collected for functional experiments. Interference efficiency was detected through qRT-PCR.

### Cell viability assay

A total of 2000 cells were plated in 96-well plates, and 10 µL of CCK-8 was added to each well. The cells were incubated in an incubator for 1 h. The wavelength was set at 450 nm. The IC_50_ values of sunitinib and sorafenib were detected at 24, 48, and 72 h. Drugs were purchased from MedChemExpress (Monmouth Junction, NJ, United States).

### Colony formation assay

Cells at the logarithmic growth stage were collected, and 1000 cells were plated in six-well plates. The cells were cultured for 10 days, fixed with methanol for 20 min, and stained with 0.1% crystal violet for 15 min. The number of clones that formed in each well was counted and photographed.

### Transwell assay

A total of 600 μL of medium containing 10% serum was added into the lower chamber of a 24-well plate. Cells were added into the upper chamber and incubated in the incubator for 24 h. The liquid in the upper chamber was aspirated dry, and the cells were fixed with formaldehyde for 20 min and stained with 0.1% crystal violet for 15 min. After air drying, a microscope was used for observation and photography. Invasion experiments required a layer of Matrigel in the upper chamber.

### Statistical analysis

Survival analysis was performed through the K–M method. The Wilcoxon signed-rank test was used to analyze DEGs, and paired *t*-test was conducted to analyze gene expression in the clinical samples. Statistical analysis was performed by using R4.0.2 and GraphPad Prism 8.0.2. *p* < 0.05 was considered a significant difference.

## Result

### Genetic variations and expression levels of m7G regulators in KIRC

**FIGURE 1:**
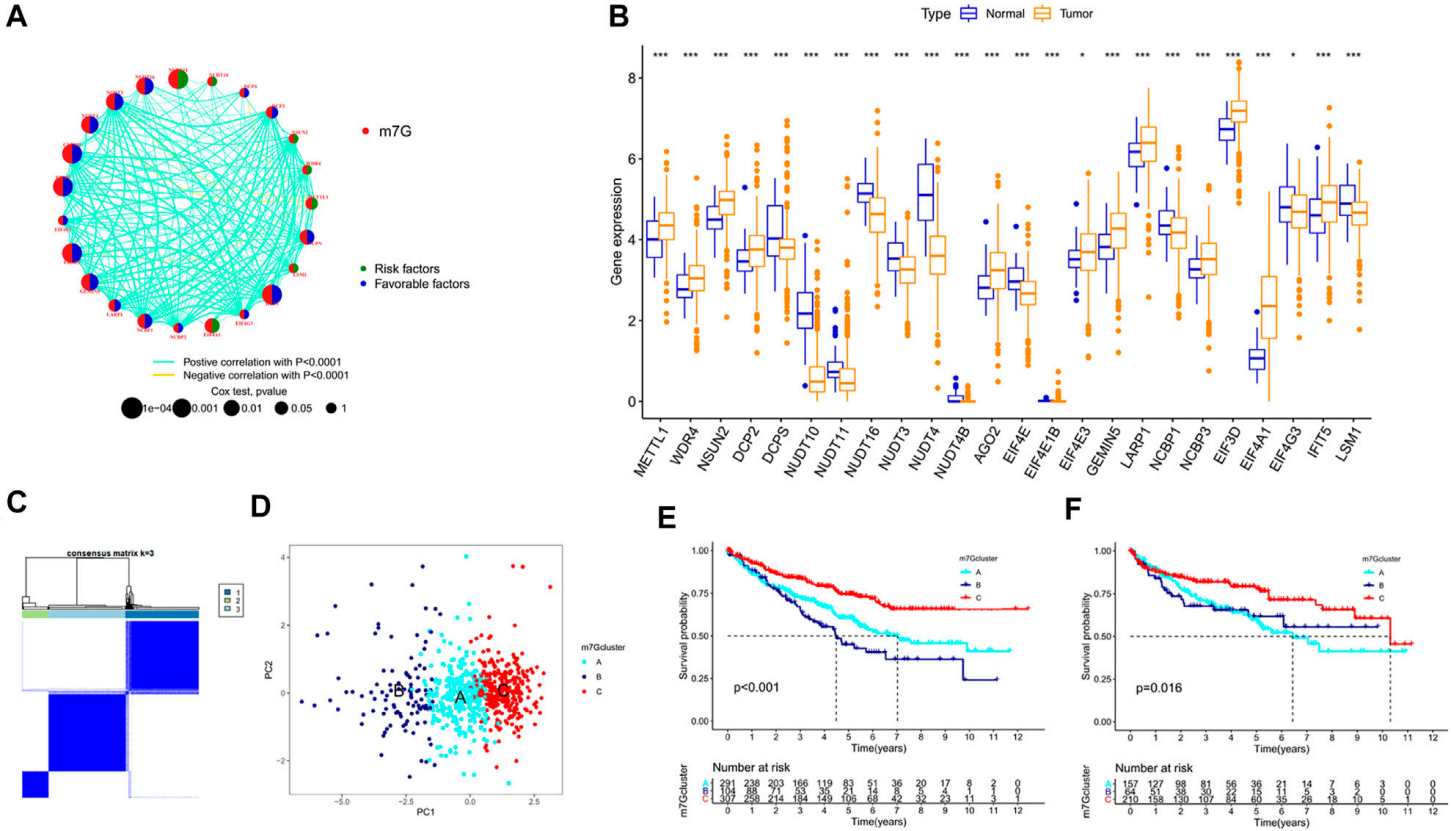
Unsupervised clustering analysis of m7G regulators in KIRC. **(A)** Interaction network of m7G regulators. **(B)** Expression levels of m7G regulators in KIRC. **(C)** Heat maps of the consensus clustering matrix. **(D)** PCA distinguishing the three subtypes. **(E–F)** K–M curves of OS and DFS in the three subtypes. KIRC, kidney renal clear cell carcinoma; OS, overall survival; DFS, disease-free survival; K–M, Kaplan–Meier.

Among the 29 m7G regulators analyzed in this study, LARP1 had the highest mutation frequency (Supplementary Figure S1). We found a significant correlation among 23 genes. METTL1 was negatively correlated with DCPS, NUDT3, NUDT4, and EIF4E3 and positively correlated with other genes. Most genes were favorable factors for patients with KIRC, whereas NUDT11, NUDT10, NSUN2, WDR4, METTL1, LSM1, and EIF4A1 were risk factors (Figure 1A). We found significant differences in 24 genes between cancer and normal tissues in the TCGA–KIRC cohort (Figure 1B). These results suggested that m7G may play an important role in KIRC.

### Expression levels of m7G regulators in KIRC

We combined the TCGA–KIRC, GSE29609, GSE49012, and E-MTAB-1980 cohorts to obtain a total of 702 samples to further understand the possible role of m7G in KIRC. Through unsupervised clustering, the samples were divided into three subtypes for analysis (Figure 1C). A total of 291, 104, and 307 cases were included in clusters A, B, and C, respectively. PCA revealed that the m7G regulator can well-distinguish the samples of each cluster (Figure 1D). Significant differences were found in overall survival (OS) and disease-free survival (DFS) among the three subtypes. The survival of cluster C was longer than that of clusters A and B (Figures 1E,F). m7G regulator expression was higher in cluster C than in other clusters (Supplementary Figure S2A).

### Pathways between different subtypes

**FIGURE 2:**
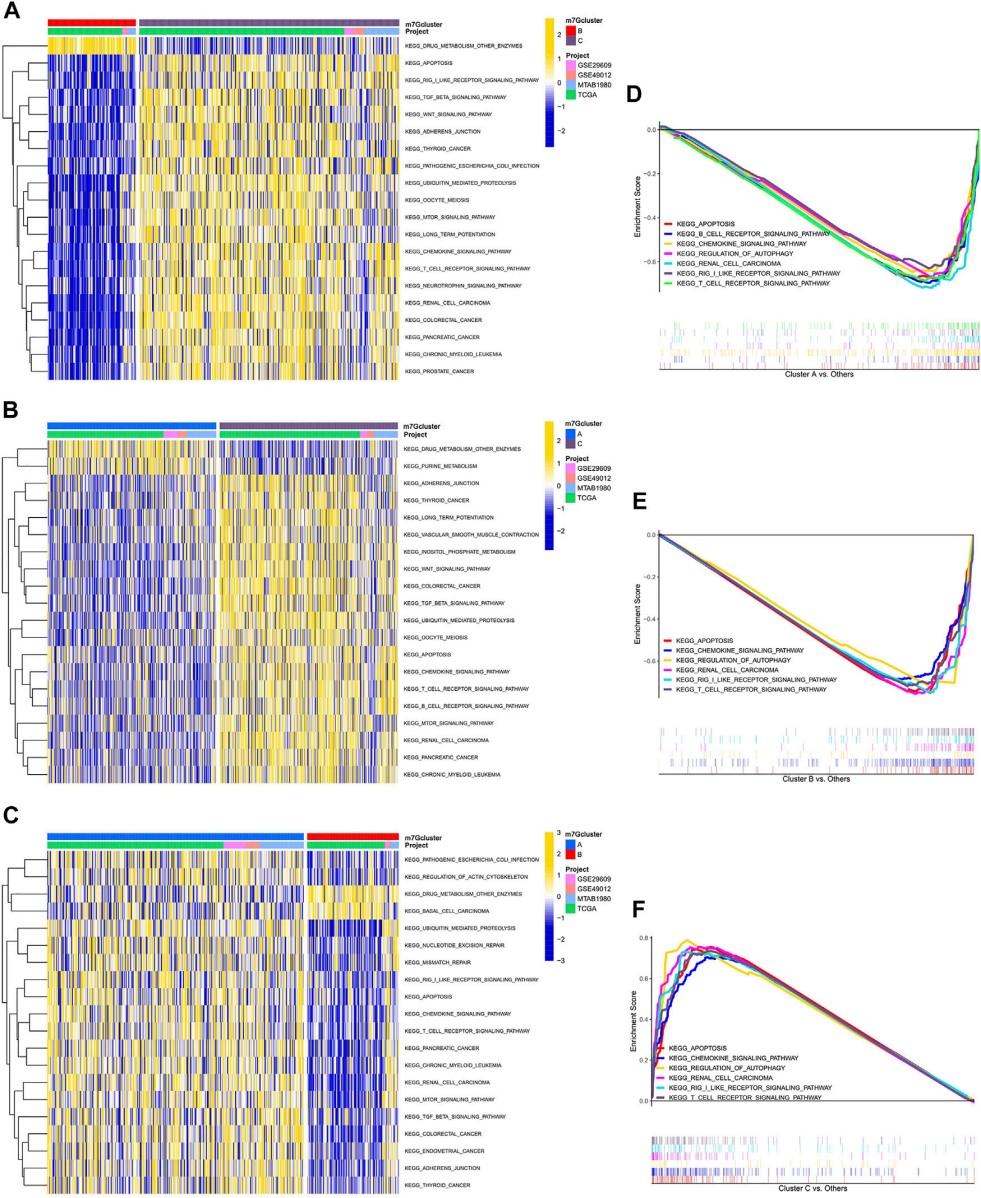
Enrichment analysis among different subtypes. **(A)** Pathway enrichment analysis between m7G clusters B and C. **(B)** Pathway enrichment analysis between m7G clusters A and C. **(C)** Pathway enrichment analysis between m7G clusters A and B. **(D–F)** Gene enrichment analysis among different subtypes.

GSVA enrichment analysis showed that immune-related pathways, such as the RIG-I-like receptor signaling pathway, the chemokine signaling pathway, the T-cell receptor signaling pathway, and apoptosis, were significantly active in the m7G cluster C (Figure 2A). Drug metabolism-related pathways were significantly active in m7G cluster B, and purine metabolism was significantly active in m7G cluster A (Figures 2B,C). In addition, RCC was significantly active in m7G cluster C. GSEA also demonstrated that immune-related pathways and apoptosis were enriched in the m7G cluster C. Autophagy was also significantly enriched in m7G cluster C (Figures 2D–F). These results can account for better prognosis of m7G cluster C than that of m7G clusters A and B.

### Differences in the immune microenvironments of the m7G clusters

**FIGURE 3:**
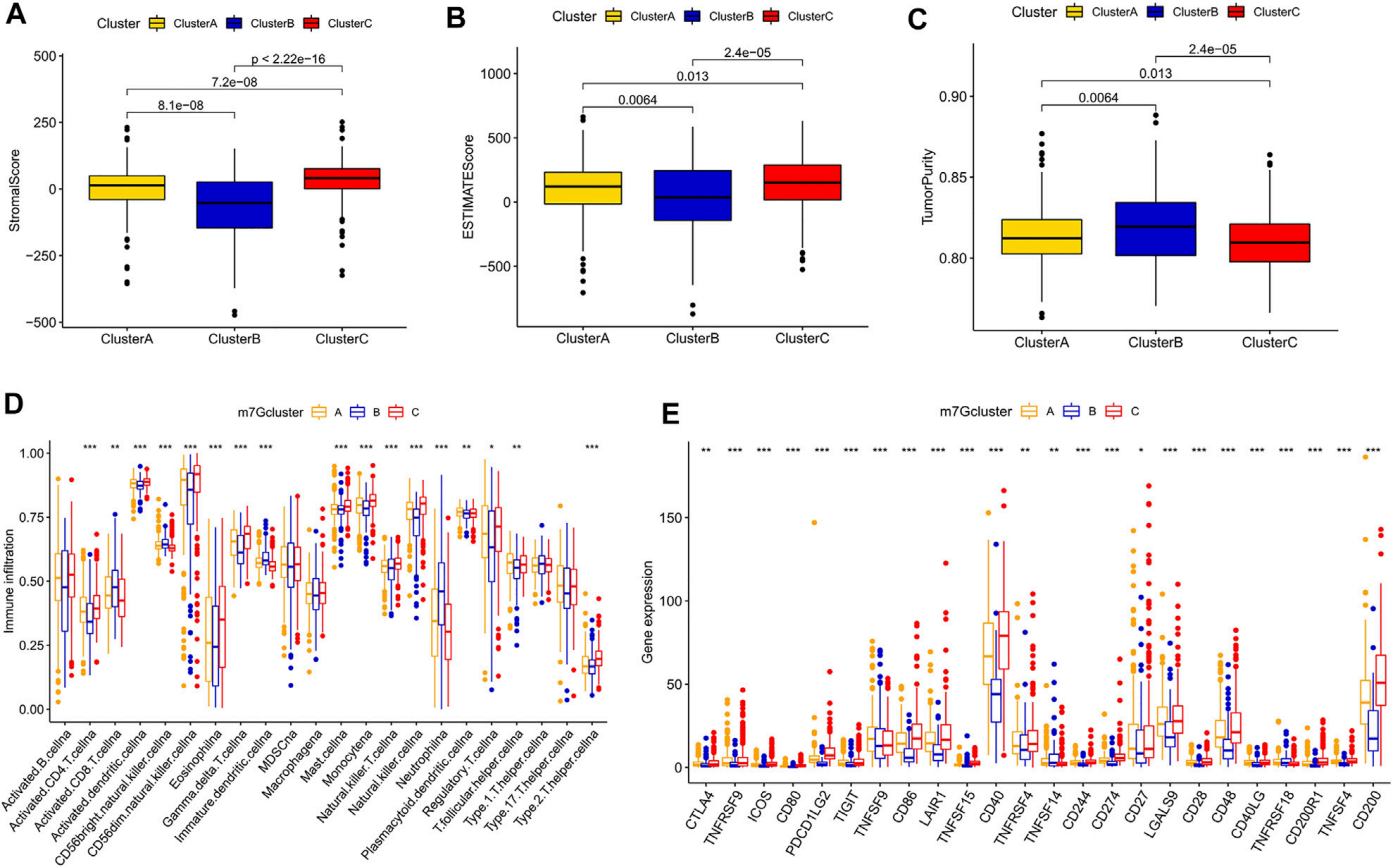
Differences in the immune microenvironment among different subtypes. **(A–C)** Differences in stromal and ESTIMATE scores and tumor purity among different subtypes. **(D)** Differences in immune cell infiltration among different subtypes based on ssGSEA. **(E)** Differences in immune checkpoint expression among different subtypes.

After scoring the tumor microenvironment by using the ESTIMATE algorithm, we found differences in stromal scores, ESTIMATE scores, and tumor purity among m7G clusters A, B, and C. m7G cluster C had higher stromal and estimate scores and lower tumor purity than the other groups (Figures 3A–C). We used ssGSEA to evaluate the degree of immune cell infiltration in the three groups. Most immune cells differed among the three subtypes. Activated CD4^+^ T cells, activated dendritic cells, CD56 dim natural killer cells, eosinophil, gamma delta T cells, mast cells, monocytes, natural killer T cells, natural killer cells, regulatory T cells, and type 2 T helper cells had higher distributions in m7G cluster C than in the other clusters, whereas activated CD8^+^ T cells, CD56 bright natural killer cells, immature dendritic cells, and neutrophils had lower distributions in m7G cluster C than in other clusters (Figure 3D). In addition, higher expression levels of immune checkpoints (Figure 3E) and chemokines (Supplementary Figure S2B) were found in m7G cluster C than in other clusters. Interestingly, in the m7GHub database, we found that 15 immune checkpoints have m7G methylation modification (Supplementary Table S3).

### Development and validation of the m7G score

**FIGURE 4:**
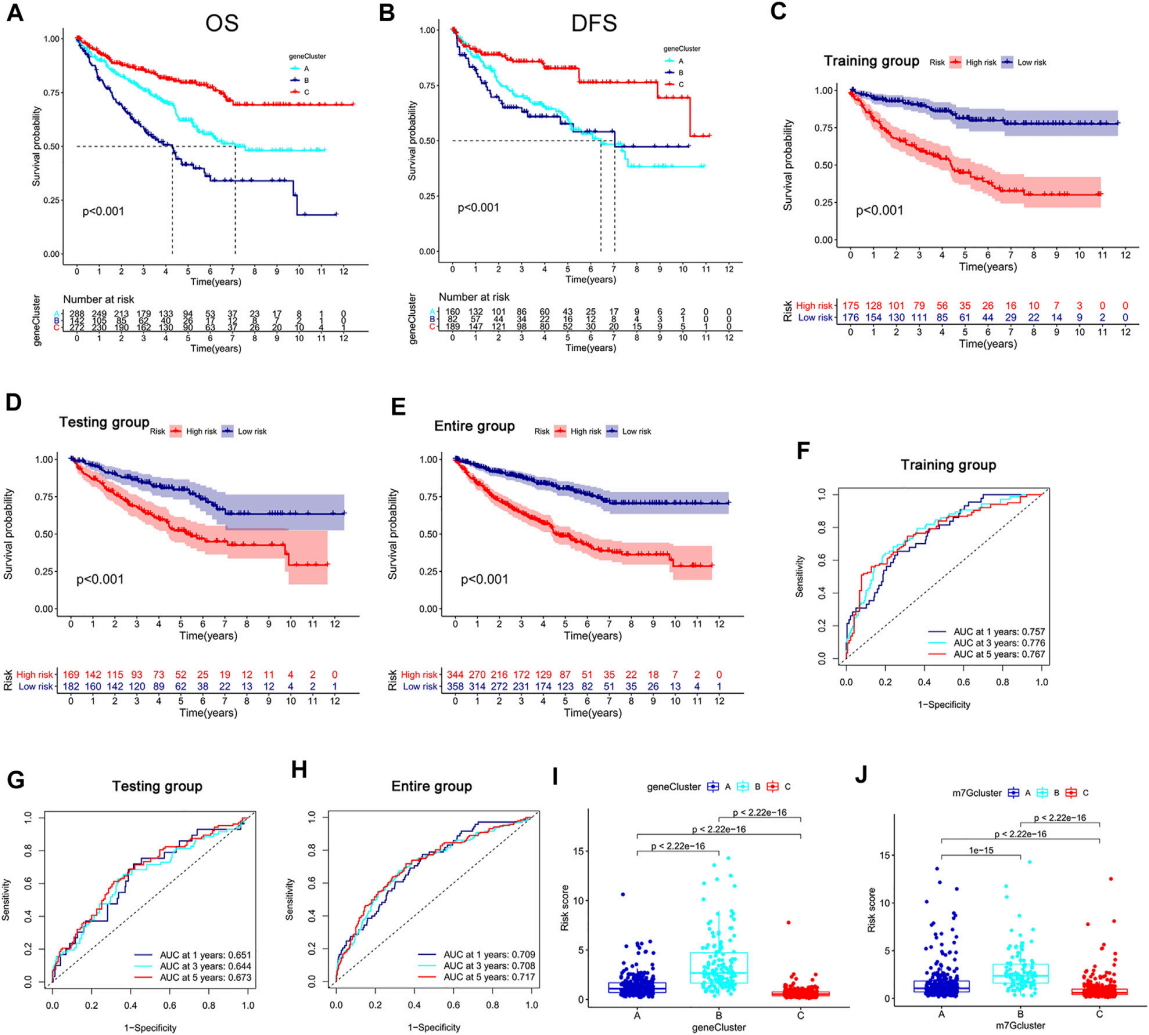
Development and validation of the m7G score. **(A,B)** K–M analysis of OS and DFS among different gene subtypes. **(C–E)** K–M survival analysis between high- and low-risk groups in the training and testing groups and the entire group. **(F–H)** ROC curve evaluating the prediction accuracy of the m7G score in the training and testing groups and the entire group. **(I,J)** Risk scores of different genes and m7G clusters. K–M, Kaplan–Meier; OS, overall survival; DFS, disease-free survival; ROC, receiver operator curve.

We obtained 80 DEGs among m7G subtypes to explore their potential biological functions in different subtypes. GO and KEGG analyses revealed that the DEGs were enriched in cancer-related pathways, such as the positive regulation of cell adhesion, the positive regulation of vasculature development, cell molecules, the PI3K–Akt signaling pathway, and the JAK−STAT signaling pathway (Supplementary Figures S3A,B). m7G modification may play a key role in carcinogenesis in KIRC. A total of 75 prognostic genes were identified through univariate Cox analysis (Supplementary Table S4). We performed unsupervised clustering of prognostic genes to obtain three gene subtypes (Supplementary Figure S3C). Significant differences in OS and PFS existed among gene clusters A, B, and C (Figures 4A,B). A total of 702 samples were divided into the training group (n = 351) and the testing group (n = 351), and the formula used to develop the m7G score was obtained after LASSO regression analysis as follows: m7G score = (−0.222 × G3BP2) + (0.195 × THBS1) + (−0.224 × BCL2) + (−0.509 × PTPRB) + (0.284 × CD36) + (−0.124 × PDK4) + (−0.187 × TMEM125). The patients were divided into high- and low-risk groups in accordance with the median value of the training group. The prognosis of the patients in the high-risk group was poorer than that of the low-risk group (Figures 4C–E). The area under the curve (AUC) showed that the m7G score has good prediction accuracy (Figures 4F–H). A Sanggi diagram was used to depict the interrelationships between the m7G cluster, gene cluster, risk, and patient survival status (Supplementary Figure S4). Significant differences in m7G scores were found between gene and m7G subtypes, and the scores of m7G cluster C and gene cluster C were lower than those of other clusters (Figures 4I,J).

**FIGURE 5:**
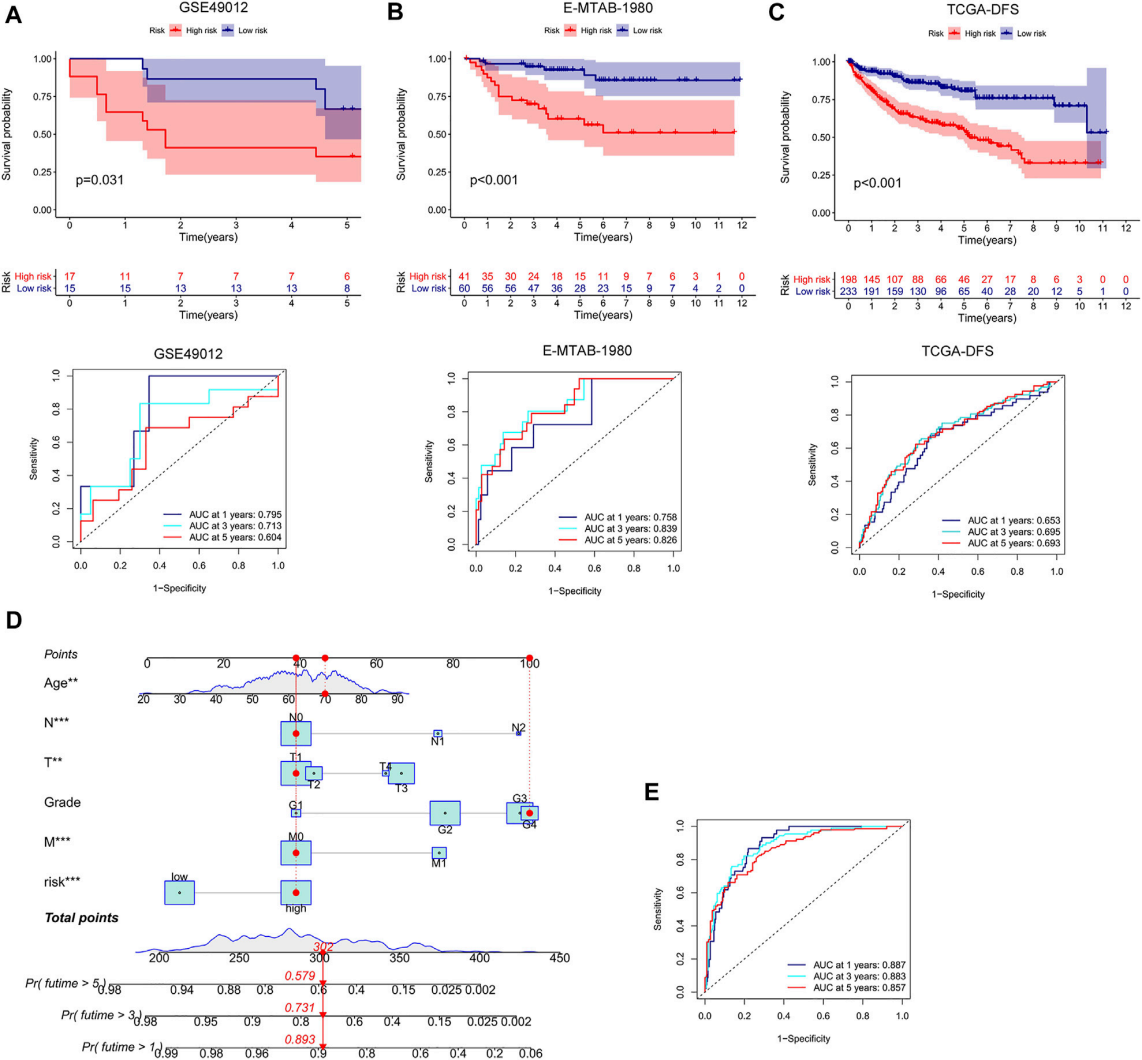
External validation and nomogram construction of the m7G score. **(A,B)** K–M analysis and ROC curve of the m7G score for the GSE40912 and E-MTAB-1980 datasets. **(C)** K–M analysis of the DFS of the TCGA–KIRC cohort. **(D)** Nomogram combining clinicopathological variables and risk score. **(E)** ROC curve was used to evaluate the accuracy of the normogram in predicting survival. K–M, Kaplan–Meier; DFS, disease-free survival; ROC, receiver operator curve; KIRC, kidney renal clear cell carcinoma.

We conducted external validation to prove the applicability of the m7G score. The m7G score still had good prediction performance on the GSE40912 and E-MTAB-1980 datasets. The prognosis of the patients in the low-risk group was better than that of the patients in the high-risk group. AUC analysis showed that the m7G score has a high prediction accuracy (Figures 5A,B). In addition, the m7G score had good predictive performance for DFS in the TCGA–KIRC cohort. A significant difference was found between high- and low-risk groups, and the survival of the patients in the low-risk group was better than that of the patients in the high-risk group (Figure 5C). The nomogram that was constructed in combination with clinicopathological variables can well-predict the survival of patients. The 1-, 3-, and 5-year AUCs reached 0.887, 0.883, and 0.857, respectively, with high prediction accuracy (Figures 5D,E).

### TME characteristics in the high- and low-risk groups

**FIGURE 6:**
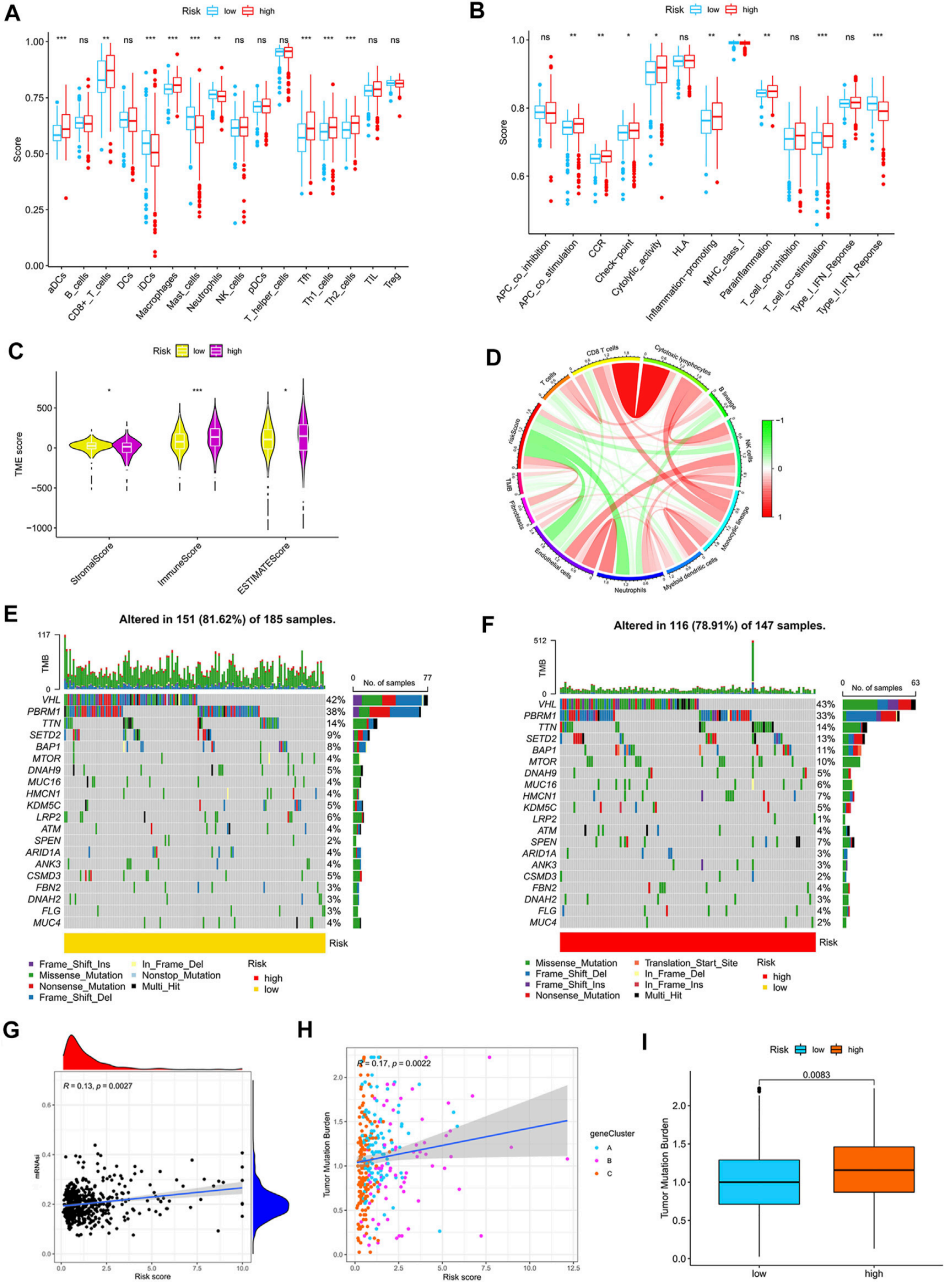
Relationship between the m7G score and tumor immune microenvironment. **(A,B)** ssGSEA of the correlation of the m7G score with immune cells and immune function. **(C)** ESTIMATE algorithm analysis of the correlation of the m7G score with immune cells and immune function. **(D)** MCP counter algorithm analysis of the correlation between the m7G score and immune cells. **(E,F)** Mutation characteristics of the high- and low-risk groups. **(G)** Correlation analysis between the m7G score and stemness indices. **(H,I)** Correlation analysis between the m7G score and TMB. TMB, tumor mutation burden.

By using the CIBERSORT algorithm, we found that the m7G score was negatively correlated with resting dendritic cells, M1 macrophages, and gamma delta T cells (Supplementary Figure S5A) and positively correlated with M0 macrophages, M2 macrophages, and neutrophils (Supplementary Figure S5B). The ssGSEA algorithm showed that aDCs, CD8^+^ T cells, macrophages, Tfh, Th1 cells, and Th2 cells had higher scores in the high-risk group than in the low-risk group. Moreover, iDCs, mast cells, and neutrophils had higher scores in the low-risk group than in the high-risk group (Figure 6A). The scores of APC co-stimulation, CCR, check-point, cytolytic activity, inflammation promotion, para-inflammation, and T cell co-stimulation were higher in the high-risk group than in the low-risk group, whereas the scores of MHC-class Ⅰ and type-Ⅱ IFN response were higher in the low-risk group than in the high-risk group (Figure 6B). We used the ESTIMATE algorithm to score the tumor microenvironment and found differences among stromal, immune, and estimate scores. The estimate score of the high-risk group was higher than that of the low-risk group (Figure 6C). The MCP counter algorithm showed that the m7G score was positively correlated with B lineage and fibroblasts and negatively correlated with NK cells, monocytic lineage, myeloid dendritic cells, neutrophils, and endothelial cells (Figure 6D).

### Correlation analysis of the m7G score with mutation, TMB and stem cell index

The mutation frequency of VHL was highest in the two groups, and the mutation frequency of mTOR was higher in the high-risk group (Figures 6E,F). The m7G score was positively correlated with stemness indices and TMB (Figures 6G,H). The higher TMB of the high-risk group than that of the low-risk group (Figure 6I) suggested that immunotherapy was more effective in high-risk patients than in low-risk patients.

### Functional mechanism analysis of high- and low-risk groups

GSEA revealed that immune- and metabolism-related pathways were significantly enriched in the low-risk groups, such as the B-cell receptor signaling pathway, the T-cell receptor signaling pathway, the chemokine signaling pathway, endocytosis, fatty acid metabolism, fructose and mannose metabolism, glycolysis, gluconeogenesis, histidine metabolism, and pyruvate metabolism (Supplementary Figure S6A).

### Correlation analysis between the m7G score and therapeutic drugs

**FIGURE 7:**
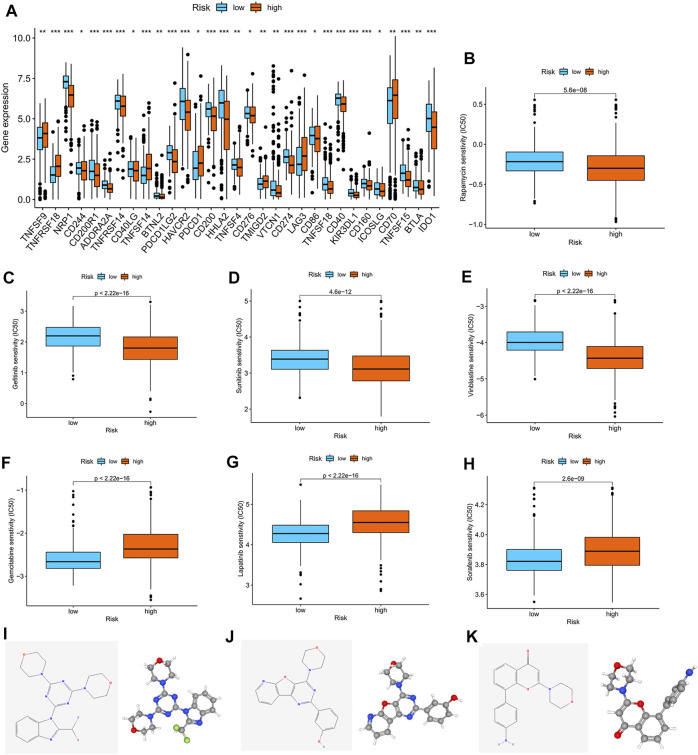
Correlation analysis between the m7G score and therapeutic drugs. **(A)** Expression profiles of checkpoints in the high- and low-risk groups. **(B–H)** Correlation analysis between the m7G score and IC_50_ of rapamycin, gefitinib, sunitinib, vinblastine, gemcitabine, lapatinib, and sorafenib. The 2D and 3D structures of **(I)** ZSTK-474, **(J)** PI-103, and **(K)** PI-828.

We found that checkpoints significantly differed between the high- and low-risk groups, and the expression of PDCD1 was higher in the high-risk group than in the low-risk group (Figure 7A). The two groups also had significantly different chemokine and chemokine receptor expression profiles (Supplementary Figure S6B). Rapamycin, gefitinib, sunitinib, and vinblastine had lower IC_50_ values in the high-risk group than in the low-risk group (Figures 7B–E), and gemcitabine, lapatinib, and sorafenib had higher IC_50_ values in the high-risk group than in the low-risk group (Figures 7F–H).

Furthermore, we used the cAMP database to explore potential small-molecule drugs for the therapy of patients with KIRC. The top 10 compounds with the strongest negative correlation with the patients in the high-risk group are shown in Table 1. Among these compounds, ZSTK-474, PI-103, and PI-828 are PI3K inhibitors. The 2D and 3D structures of the three compounds are shown in Figures 7I–K.

**TABLE 1:** Ten most negatively correlated small-molecule compounds screened from the CMap database.

Score	CMap name	Target	MOA
−96.37	Calyculin	PPP1CA, PPP1CC, and PPP2CA	Protein phosphatase inhibitor
−96.48	TG-101348	JAK2, FLT3, BRD4, JAK1, JAK3, RET, TYK2	FLT3 inhibitor, JAK inhibitor
−96.93	AZD-7762	CHEK1, and CHEK2	CHK inhibitor
−97.18	CS-110266	SLC6A3	Dopamine receptor agonist
−97.42	PI-828		PI3K inhibitor
−97.6	PI-103	PIK3CA, PIK3CG, MTOR, PIK3CB, PIK3CD, and PRKDC	MTOR inhibitor, PI3K inhibitor
−97.71	RO-90-7501	APP	Beta amyloid inhibitor
−97.77	Naftopidil	ADRA1A and ADRA1D	Adrenergic receptor antagonist
−97.8	PJ-34	EEF2, PARP1, PARP15, and PARP3	PARP inhibitor
−98.41	ZSTK-474	PIK3CG, PIK3CA, PIK3CB, and PIK3CD	PI3K inhibitor

MOA: Mechanisms of action.

### Validation in clinical samples

**FIGURE 8:**
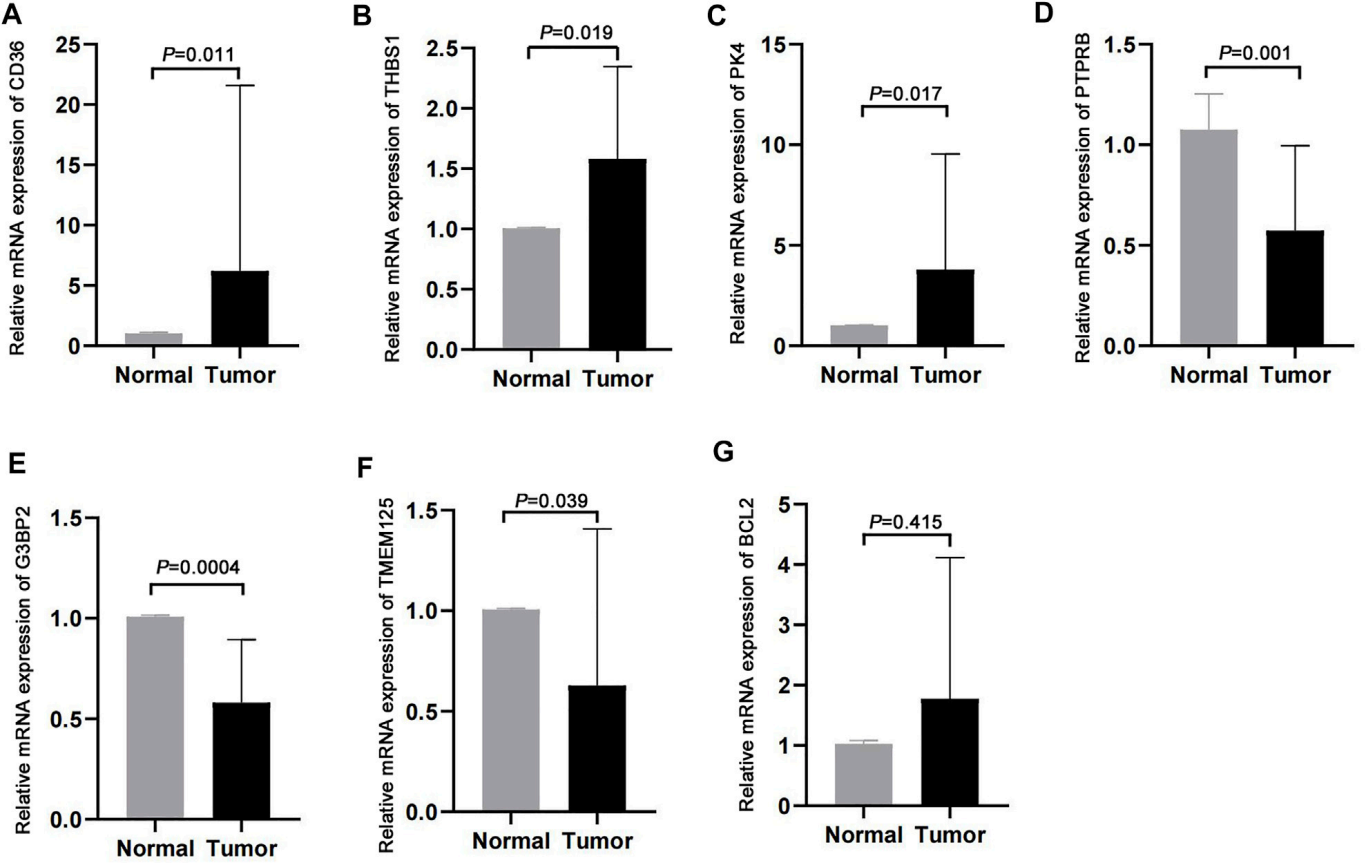
Validation in clinical samples. **(A–G)** Expression of CD36, THBS1, PDK4, PTPRB, G3BP2, TMEM125, and BCL2 in cancer and normal tissues.

We validated the expression profiles of the genes that were used to develop the m7G score in paired cancer and normal tissues. CD36, THBS1, and PDK4 were highly expressed in cancer tissues, whereas PTPRB, G3BP2, and TMEM125 were lowly expressed in cancer tissues (Figures 8A–F). However, we found no difference in the expression of BCL2 between cancer and normal tissues (Figure 8G).

### Effects of WDR4 on the biological function and drug resistance of KIRC cells

**FIGURE 9:**
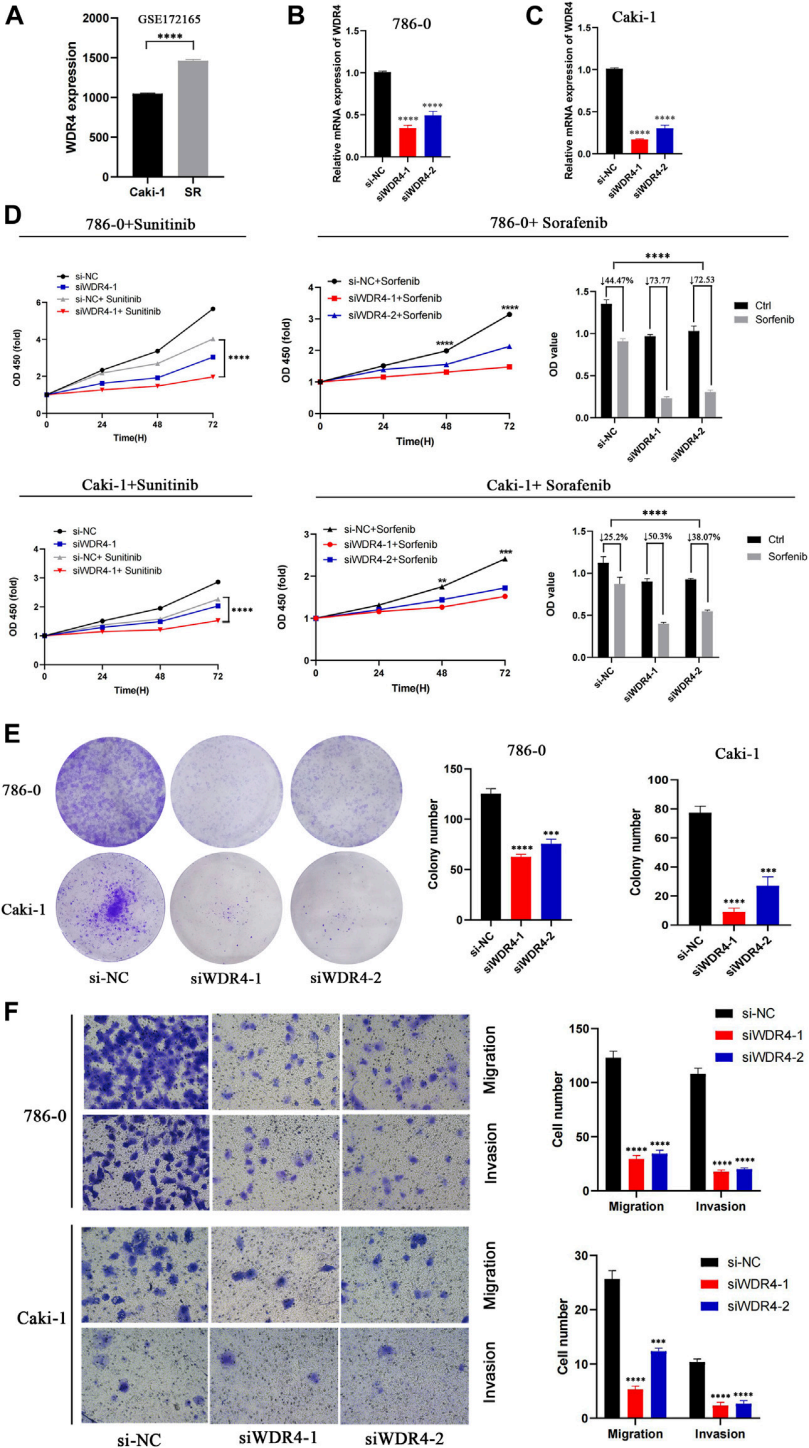
Effects of WDR4 on the biological function and drug resistance of KIRC cells. **(A)** Expression of WDR4 in sunitinib-resistant cell lines. **(B–C)** Detection of WDR4 knockdown efficiency. **(D)** Effects of WDR4 knockdown on KIRC cell proliferation and drug sensitivity. **(E)** Effect of WDR4 knockdown on the colony formation ability of KIRC cells. **(F)** Effects of knockdown of WDR4 on the migration and invasion of KIRC cells. SR: sunitinib-resistant.

In the GSE172165 dataset, WDR4 was significantly upregulated in the sunitinib-resistant Caki-1 cell line (Figure 9A). The anticancer drugs with the highest correlation with WDR4 in the GDSC database are shown in Table 2. WDR4 was positively correlated with the IC_50_ values of lapatinib, erlotinib, entinostat, and sorafenib. We further experimentally verified the effect of WDR4 on sensitivity to sunitinib and sorafenib, which are common chemotherapeutic drugs for KIRC. We used siRNA to knock down WDR4 in 786-0 and Caki-1 cells. The qRT-PCR results showed that interference with WDR4 significantly reduced the expression of WDR4 (Figures 9B,C). The knockdown of WDR4 decreased the cell viability and the IC_50_ values of sunitinib and sorafenib in 786-0 and Caki-1 cells (Figure 9D). Colony-formation assays demonstrated that the knockdown of WDR4 significantly inhibited the proliferation of 786-0 and Caki-1 cells (Figure 9E). The Transwell assay illustrated that knocking down WDR4 could significantly inhibit the migration and invasion of 786-0 and Caki-1 cells (Figure 9F). These results suggested that WDR4 may be a potential therapeutic target in patients with KIRC and sunitinib and sorafenib resistance.

**TABLE 2:** Four most correlated anticancer drugs screened from the GDSC database.

Gene	Drug	Cor	Label	*p* Value
WDR4	Lapatinib	0.587	Positive	0.000336
WDR4	Erlotinib	0.545	Positive	0.000863
WDR4	Entinostat	0.531	Positive	0.001175
WDR4	Sorafenib	0.526	Positive	0.001310

GDSC: Genomics of drug sensitivity in cancer.

## Discussion

KIRC is highly heterogeneous, and the evaluation of its prognosis is dependent on TNM staging (Pichler et al., 2013). Reliable biomarkers for predicting the prognosis of KIRC are lacking. Molecular subtype predictors can be used to predict prognosis and response to immunotherapy and provide a basis for the precise treatment of patients with cancer. Exploring the molecular regulatory mechanisms of different subtypes has become a research hotspot in the field of cancer. m6A is the most common RNA methylation modification, and the molecular subtype based on m6A regulators can well-predict the efficacy of immunotherapy and evaluate the prognosis of patients (Shen et al., 2020; Zhang et al., 2020; Zhong et al., 2021). 5mC, another type of methylation modification, can also well-predict the prognosis of patients (Hu Y et al., 2021).

In our research, we found that most m7G regulators were favorable factors for KIRC, whereas NUDT11, NUDT10, NSUN2, WDR4, METTL1, LSM1, and EIF4A1 were risk factors. These genes all play an oncogenic role in cancer (Chen Z et al., 2021; Grisanzio et al., 2012; Hu J et al., 2021; Little et al., 2016; Wang et al., 2021; Xia et al., 2021; Ying et al., 2021; Zhao et al., 2021). Pan-cancer analysis showed that WDR4 and METTL1 were closely related to cancer immune infiltration and were immunotherapy targets in patients, and the high expression of KIRC was associated with poor prognosis of patients (Gao et al., 2022; Zeng et al., 2021). On the basis of the 29 m7G regulators, we divided the 702 patients with KIRC into three subtypes. We found significant differences in the OS and DFS between subtypes and that the prognosis of m7G cluster C was better than that of other clusters. m7G cluster C is related to immune activation pathways, such as RIG-I-like receptor, chemokine, and T-cell receptor signaling pathways. Cluster B is related to drug metabolism-related pathways, and cluster A is related to purine metabolism. Tumor purity and CD8^+^ T cell infiltration were lower in the m7G cluster C than in other clusters. In RCC, CD8^+^ T cells were mostly disabled and promoted immune escape. Studies have shown that in contrast to that in most solid tumors, the high infiltration of CD8^+^ T cells in RCC predicts poor survival outcomes (Fridman et al., 2017). These results can explain why m7G cluster C had better prognosis than other clusters. Checkpoint inhibitors have been approved for the first-line treatment of KIRC. In our study, most checkpoints were more highly expressed in m7G cluster C than in other clusters, thereby suggesting that m7G cluster C may benefit more from checkpoint inhibitor therapy than other clusters. DEGs among different m7G subtypes were enriched in cancer-related pathways, such as the positive regulation of cell adhesion, the positive regulation of vasculature development, cell molecule adhesion, the PI3K–Akt signaling pathway, and the AKT–STAT signaling pathway. PI3K–Akt signaling pathway activation can promote the metastasis and progression of RCC (Du et al., 2021; Lin et al., 2021; Zhu et al., 2020). We divided the patients into three gene types in accordance with the three m7G subtypes and found significant differences in OS and PFS among the subtypes. We developed a new m7G score and validated the genes in clinical samples. CD36, PDK4, and THBS1 were highly expressed in cancer tissues, and G3BP2, PTPRB, and TMEM125 were lowly expressed in cancer tissues. However, we found no difference in the expression of BCL2 between cancer and normal tissues, likely due to our small sample size. The high expression of CD36, THBS1, and PDK4 in cancer is related to poor prognosis and promotes tumor progression (Guda et al., 2018; Huang et al., 2017; Kim et al., 2019; Liu et al., 2021; Liu et al., 2020; Xu et al., 2019; Zhou et al., 2009). G3BP2 and PTPRB are lowly expressed in cancer and are reliable markers for prognosis of patients (Qi et al., 2016; Wei et al., 2015). In addition, single-cell transcriptomes showed that PTPRB was expressed in the endothelial cells of normal kidney tissue (Young et al., 2018).

A total of 702 patients were divided into training and testing groups. The m7G score can well-predict the prognosis of the patients in the training and testing groups and the entire group with high prediction accuracy. The m7G score was externally validated with the GSE40912 and E-MTAB-1980 datasets and still had a good predictive performance. Therefore, the m7G score can be used as an effective biomarker for prediction of the prognosis of patients with KIRC and is helpful in clinical treatment decision-making. In addition, for improving the prediction of the prognosis of patients, we combined the TNM stage, grade, and age to construct a nomogram. This approach improved the predictive performance of the m7G score. At the same time, we found that cluster C of the immune activation subtype had a low m7G score. The tumor microenvironment plays an important role in the development of cancer. The main function of M1 macrophages is to promote antigen presentation, secrete immune-activating factors, and play an antitumor role (Chanmee et al., 2014; Hu et al., 2016). M2-like macrophage polarization can promote the formation of an immunosuppressive microenvironment in glioma (Xu et al., 2021). NELF in CD8^+^ T cells acts on the enhancers and promoters of TCF1 target genes to exert antitumor immunity (Wu et al., 2022). The absence of a role of CD8^+^ T cells in KIRC is related to poor prognosis (Dai S et al., 2021). Fibroblasts can inhibit cancer immunity, promote cancer progression, and make patients resistant to immunotherapy, which is related to poor prognosis (Peltier et al., 2022). Our results were consistent with these findings. The m7G score was negatively correlated with M1 macrophages. M2 macrophages, CD8^+^ T cells, and fibroblasts were more abundant in the high-risk group than in the low-risk group. These immune cells were associated with the poor prognosis of patients with KIRC (Davidsson et al., 2020; Komohara et al., 2011; Li et al., 2020; Nakayama et al., 2018; Xie et al., 2021; You et al., 2021).

TMB is a reliable prognostic marker in patients with cancer, and high TMB predicts a poor prognosis (Song et al., 2022; Yang et al., 2022). High stemness indices suggest a poor prognosis and a high degree of malignancy in cancer (Zheng et al., 2021). The m7G score was positively correlated with TMB and stemness indices. The low-risk group was closely related to the immune activation pathway, which plays a role in inhibiting cancer. These results can be used to explain why the prognosis of patients with high scores is poorer than that of patients in the low-risk group. Studies have shown that the VHL mutation is the most common mutation in KIRC (Au et al., 2021). Consistent with our results, the frequency of the VHL mutation was highest in the high- and low-risk groups. Sunitinib is the first-line treatment for patients with metastatic KIRC. We found that high-risk patients were sensitive to rapamycin, gefitinib, sunitinib, and vinblastine but resistant to gemcitabine, lapatinib, and sorafenib. These findings provide a basis for personalized treatment of patients with KIRC. In addition, we found that the knockdown of WDR4 could inhibit the proliferation, migration, and invasion of 786-0 and Caki-1 cells and increase the drug sensitivity of sunitinib and sorafenib. WDR4 is a potential therapeutic target in patients with KIRC.

Our study has limitations. First, we only validated the m7G score with a small sample. Therefore, we need to validate this index with a large clinical cohort. Second, there is a lack of validation of the immunotherapy cohort, and the prediction of the m7G score for KIRC immunotherapy is limited.

## Conclusion

This study proposed a new m7G modification-related molecular subtype and illustrated the immune cell infiltration characteristics of different subtypes. The developed m7G score can well-predict the prognosis of patients with KIRC and provide a basis for their personalized treatment.

## Data Availability

The datasets presented in this study can be found in online repositories. The names of the repository/repositories and accession number(s) can be found in the article/Supplementary Material.
