# Supplementary figures and images for: m7G regulator-mediated molecular subtypes and tumor microenvironment in kidney renal clear cell carcinoma
Source: Front Pharmacol. 2022 Sep 6;13:900006. doi: 10.3389/fphar.2022.900006 (PMC9486008; doi:10.3389/fphar.2022.900006)

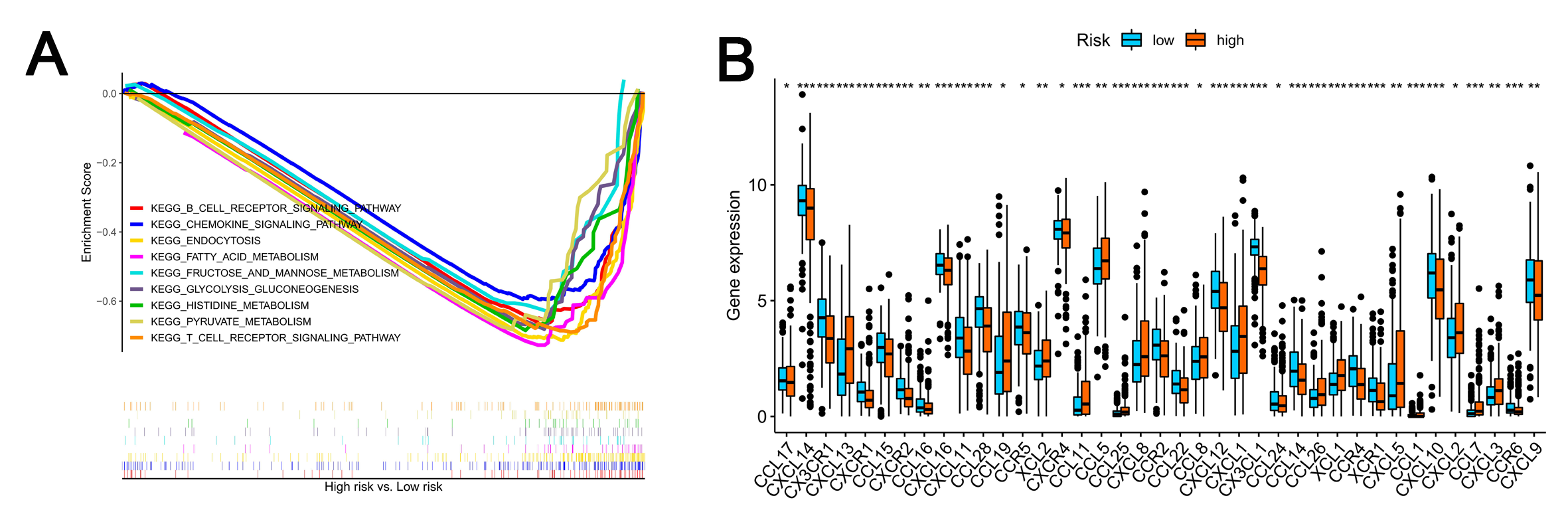

Supplement: Supplementary file 2 [file Image6.TIF]

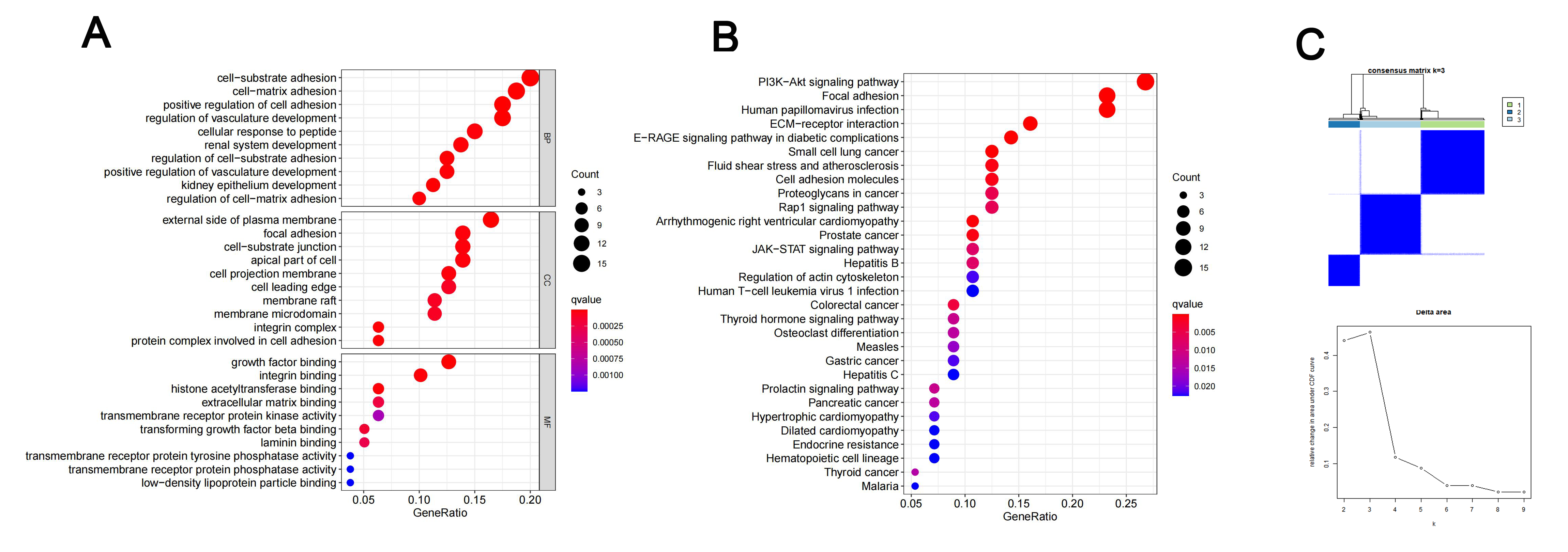

Supplement: Supplementary file 3 [file Image3.TIF]

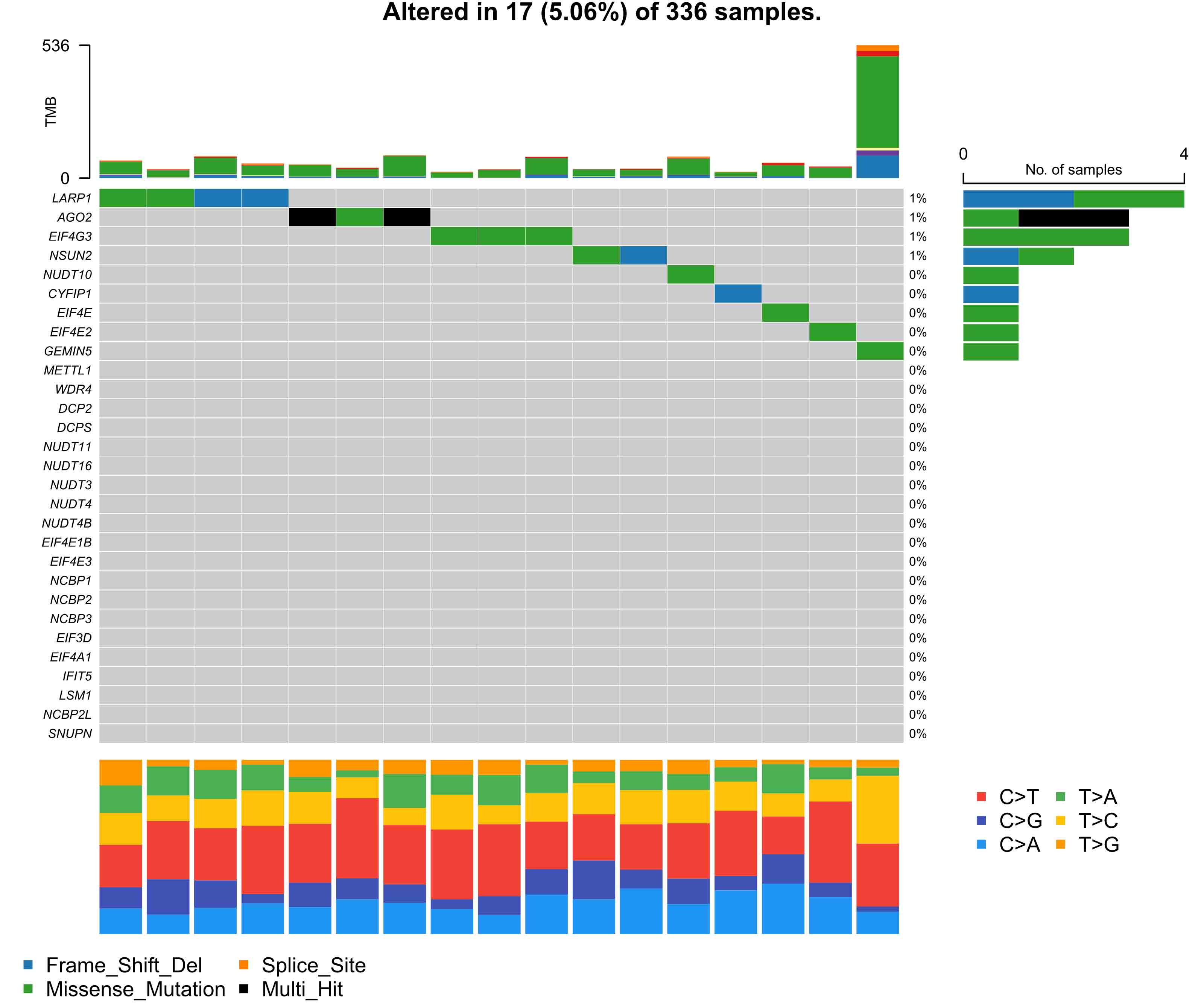

Supplement: Supplementary file 4 [file Image1.JPEG]

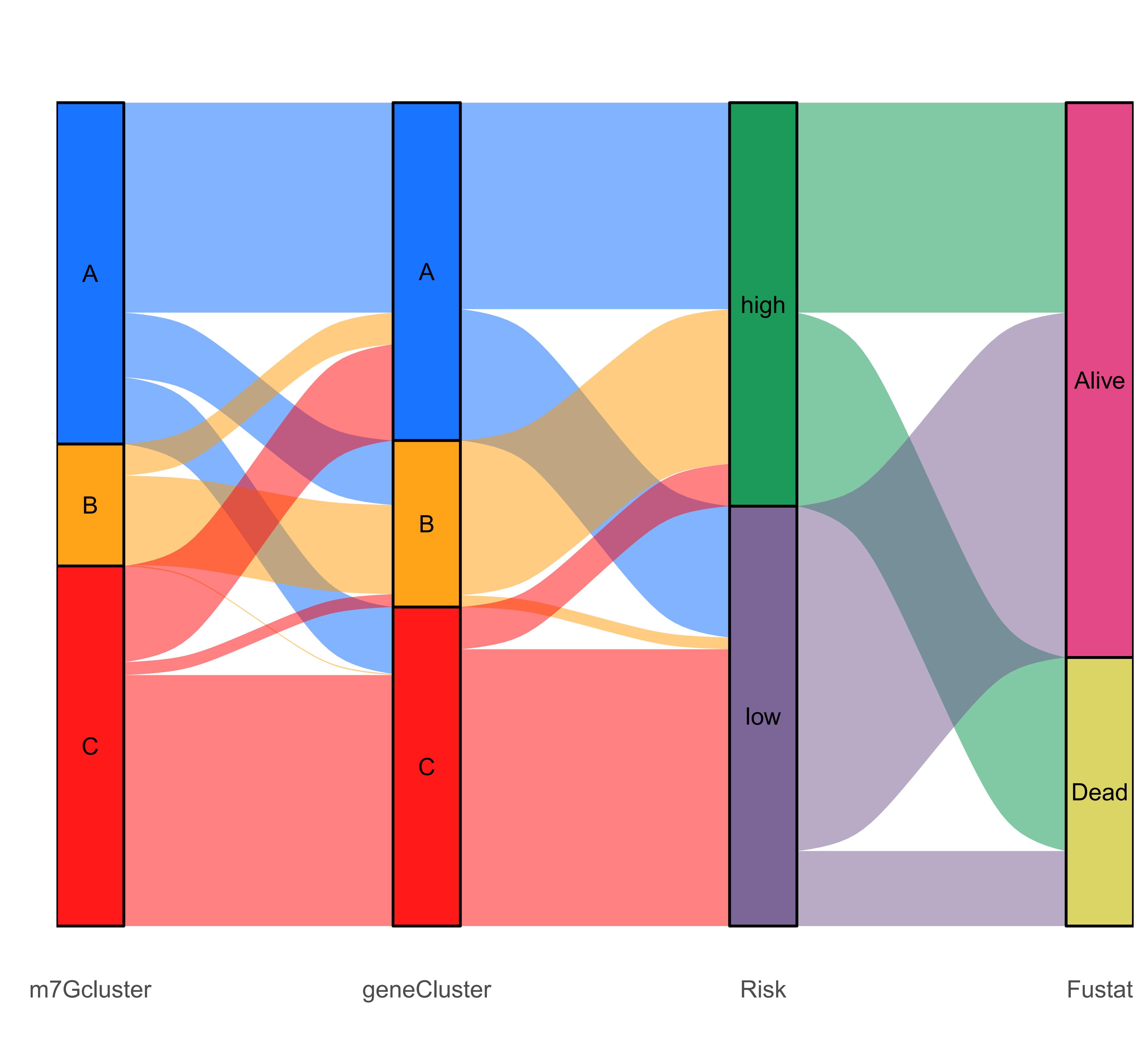

Supplement: Supplementary file 5 [file Image4.JPEG]

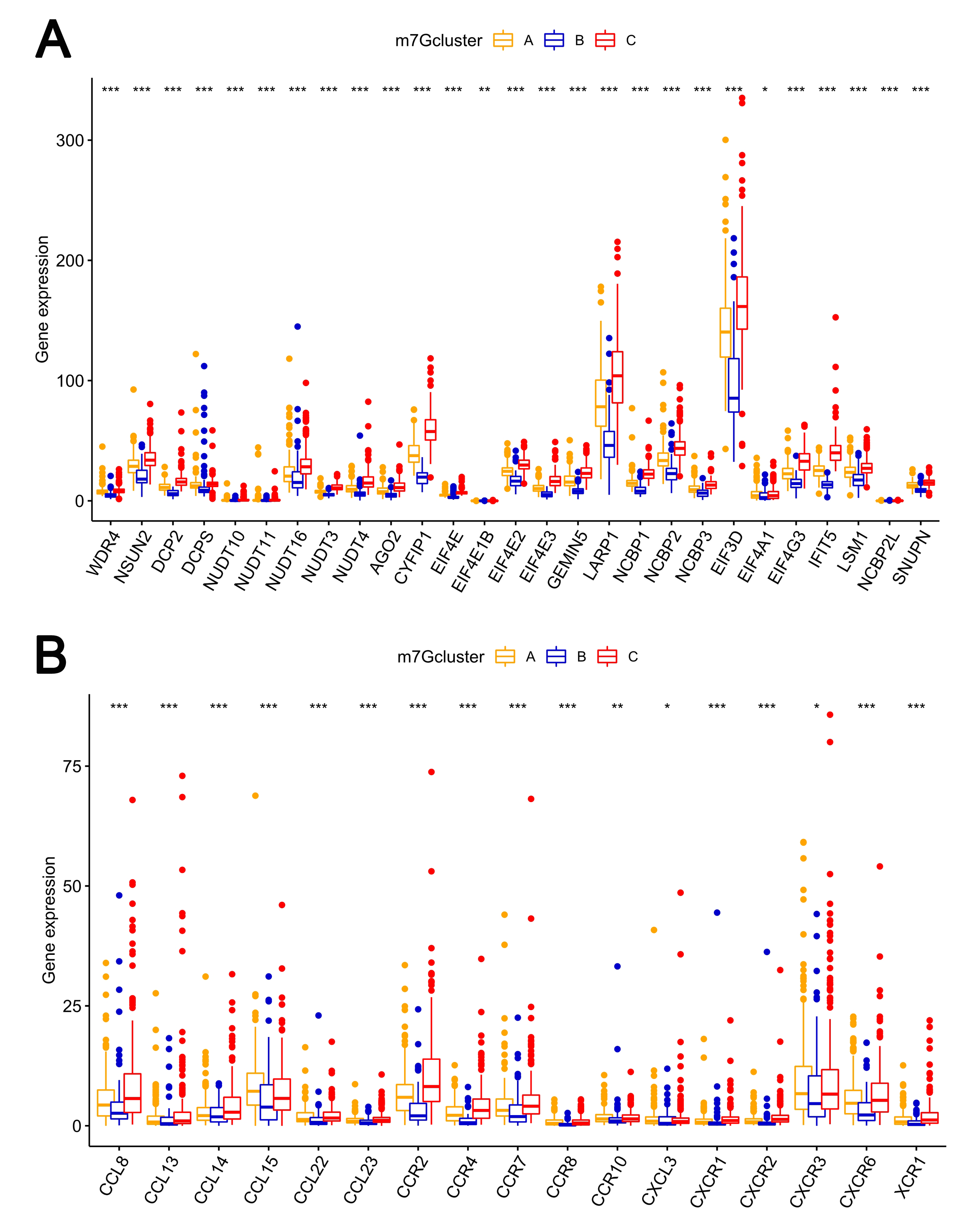

Supplement: Supplementary file 6 [file Image2.TIF]

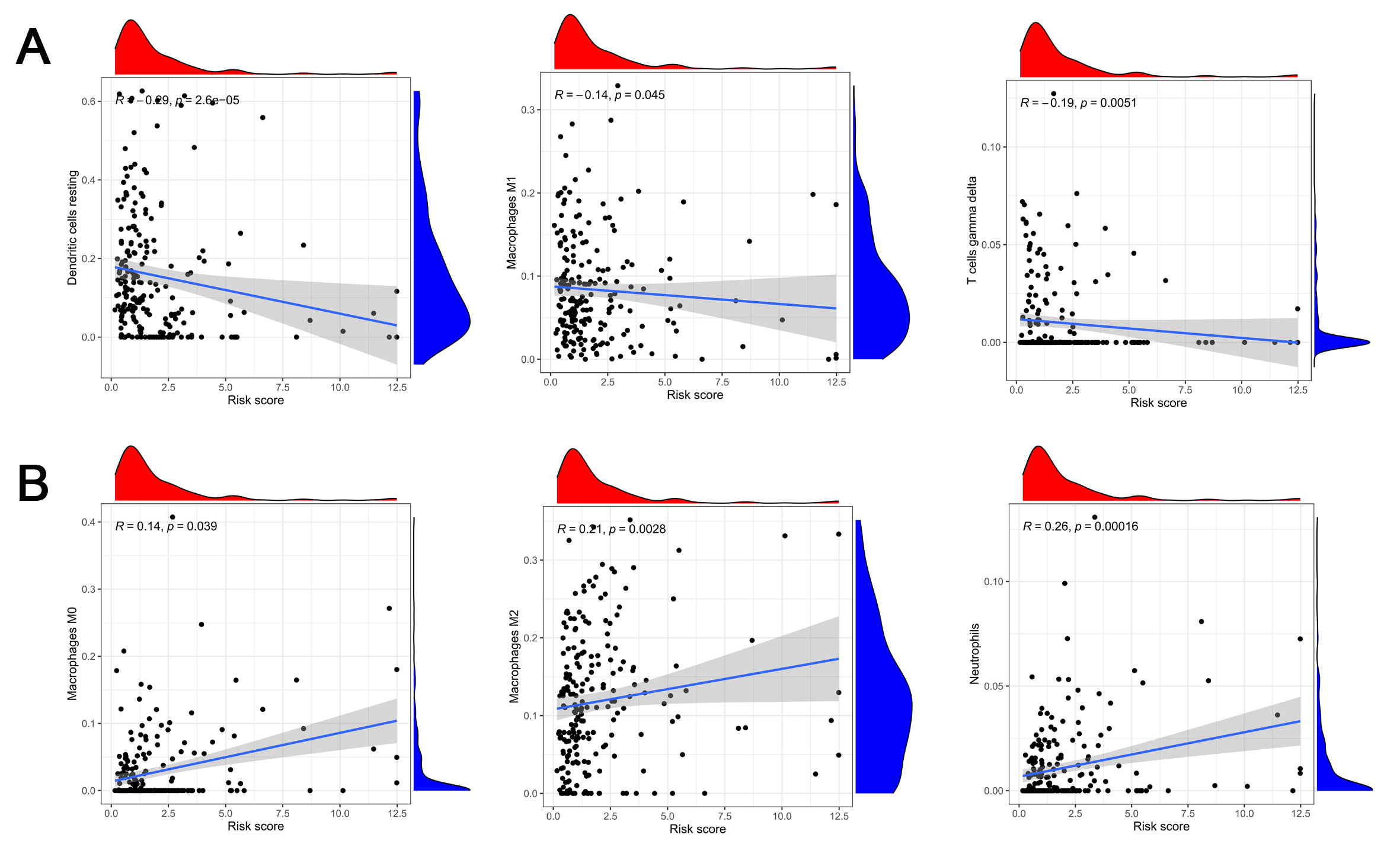

Supplement: Supplementary file 10 [file Image5.TIF]
