# Supplementary material for: m7G regulator-mediated molecular subtypes and tumor microenvironment in kidney renal clear cell carcinoma
Source: Front Pharmacol. 2022 Sep 6;13:900006. doi: 10.3389/fphar.2022.900006 (PMC9486008; doi:10.3389/fphar.2022.900006)
Supplement: Supplementary file 7 [file Table2.docx]

**Supplementary Table S2. Primer sequences for qRT-PCR**

| Gene | Forward (5’-3’) | Reverse (5’-3’) |
| --- | --- | --- |
| CD36 | CTGTTATGGGGCTATAGGGATC | ACTCCATCTGCAGTATTGTTGT |
| PDK4 | ACTCCATCTGCAGTATTGTTGT | ACACGATGTGAATTGGTTGGTCTGG |
| THBS1 | TGATGCCTGTGATGATGACGATGAC | ACTGAGCTGGGTTGTAATGGAATGG |
| G3BP2 | TGCTGAATAAAGCTCCGGAATA | TTTCCACTAGCATCTACTCCAC |
| PTPRB | GCACCCTCTCCCTTCCTACCTG | AGGCTCTCCATCTCTGCTCCAAG |
| TMEM125 | GCTGCTGTATCAAGTGGGTGTGAG | AACTGTCCTGAGATGCTGAAGATGC |
| BCL2 | GACTTCGCCGAGATGTCCAG | GAACTCAAAGAAGGCCACAATC |
| WDR4 | GCATCGAGTCCTTCTGCTTG | GATGTAGACCACAGGAGTGCC |
| GAPDH | CAGGAGGCATTGCTGATGAT | GAAGGCTGGGGCTCATTT |
